# Access to Recreational Physical Activities by Car and Bus: An Assessment of Socio-Spatial Inequalities in Mainland Scotland

**DOI:** 10.1371/journal.pone.0055638

**Published:** 2013-02-07

**Authors:** Neil S. Ferguson, Karen E. Lamb, Yang Wang, David Ogilvie, Anne Ellaway

**Affiliations:** 1 Department of Civil and Environmental Engineering, University of Strathclyde, Glasgow, Scotland, United Kingdom; 2 Medical Research Council Social and Public Health Sciences Unit, Glasgow, Scotland, United Kingdom; 3 Medical Research Council Epidemiology Unit and United Kingdom Clinical Research Collaboration Centre for Diet and Activity Research (CEDAR), Cambridge Institute of Public Health, University of Cambridge, Cambridge, England, United Kingdom; Harvard School of Public Health, United States of America

## Abstract

Obesity and other chronic conditions linked with low levels of physical activity (PA) are associated with deprivation. One reason for this could be that it is more difficult for low-income groups to access recreational PA facilities such as swimming pools and sports centres than high-income groups. In this paper, we explore the distribution of access to PA facilities by car and bus across mainland Scotland by income deprivation at datazone level. GIS car and bus networks were created to determine the number of PA facilities accessible within travel times of 10, 20 and 30 minutes. Multilevel negative binomial regression models were then used to investigate the distribution of the number of accessible facilities, adjusting for datazone population size and local authority. Access to PA facilities by car was significantly (*p<0.01*) higher for the most affluent quintile of area-based income deprivation than for most other quintiles in small towns and all other quintiles in rural areas. Accessibility by bus was significantly lower for the most affluent quintile than for other quintiles in urban areas and small towns, but not in rural areas. Overall, we found that the most disadvantaged groups were those without access to a car and living in the most affluent areas or in rural areas.

## Introduction

There is a growing body of evidence linking low levels of physical activity with obesity and a range of preventable chronic conditions, the prevalences of which are also known to increase with increasing deprivation [1]. The degree to which the built environment supports or constrains the adoption of a physically active lifestyle has emerged as an important public health consideration in recent years [2] reflecting the critical role that the design and configuration of transport and land use systems may play, alongside personal and social factors, in influencing levels of physical activity.

Physical activity (PA) may be undertaken for its own sake or in the course of doing something for another purpose, like walking or cycling to work [3]. There has been a good deal of interest in the role of the built environment in facilitating active travel, for example [4]. A related branch of research has focussed on the link between PA and access to amenities. These provide opportunities for PA in informal settings such as streets, parks and open spaces or using formal recreational facilities (for example, swimming pools, tennis courts and sports centres) which provide specialised equipment, playing surfaces or other such provision.

A significant proportion of PA currently takes place in formal recreational facilities. In Scotland, for example, whilst 81% of adults participated in at least 10 minutes of physical activity in the previous 4 weeks in 2011 (and 39% of adults met recommended PA levels), 54% of men and 45% of women reported having participated in some form of sport and exercise. This was the most common form of PA for men and the second most common form of PA for women [5]. In England, sport and exercise was a significant contributor to the total amount of PA for adults meeting minimum recommended PA levels, particularly amongst younger age groups [6]. Participation in sport in both formal and informal settings was found to range from 61% in the most affluent areas of Scotland to 42% in the most deprived areas [7]. One possible explanation for the lower participation levels in more deprived areas is that there may be lower levels of accessibility to PA facilities in poorer areas compared to more affluent areas, and that taking steps to improve access may increase participation and hence yield important health benefits.

To date, most research which has examined the association between socio-economic status and the accessibility of PA facilities has focussed on the availability of local or neighbourhood facilities, with neighbourhoods being defined using either small areal units following established administrative or statistical geographical boundaries or circular buffers. This research has been conducted in several different countries, in a variety of physical and social contexts and at different scales, and has employed different methods in sourcing and grouping facility data and in measuring accessibility. It is therefore not surprising that no clear pattern in results has emerged. Several US studies found lower socio-economic neighbourhoods to be disadvantaged in terms of access to PA facilities [8]–[11]. In contrast, lower socio-economic status neighbourhoods were observed to have better access to gyms in a study conducted in Northern California [12]. In Europe, there was no evidence that the availability of sports and recreational facilities varied by neighbourhood socio-economic status in the city of Eindhoven in the Netherlands [13] whilst, in the Paris Region of France, the direction of this relationship was found to be dependent on facility type; for example, tennis courts were found to have a higher prevalence in high median income areas whilst the opposite was the case for athletics’ facilities [14]. Finally, within the UK, a positive relationship was identified between the socio-economic status of an area and the availability of facilities in England [15], while in Scotland middle-income areas were found to have significantly better access to facilities than either low or high-income areas [16].

The accessibility of publicly-funded facilities has merited specific attention in the literature, reflecting the fact that public policy can be used to target resources on more deprived areas to provide facilities which would not otherwise be supplied by the private sector. Within one city – Glasgow, Scotland – Macintyre et al. [17] observed that public sports centres were more common in more deprived areas, and in a nationwide study across Scotland, the most affluent areas were found to contain fewer public facilities than other areas [16]. In contrast, in England, Hillsdon et al. [15] found a negative association between area deprivation index and the mean number of public facilities per head of population.

One limitation of the aforementioned studies is that any facility located outside the boundary of an arbitrarily defined area is deemed to be inaccessible. Some studies have used the road network distance to the nearest facility to measure accessibility [17]–[18]. A more comprehensive approach was adopted in Perth, Australia in which gravity-model-based accessibility measures were calculated for a variety of facility types using a road network distance decay function to weight facilities [19]. In this study, low socio-economic status areas were found to enjoy better access to gyms, sports centres and swimming pools but not golf courses.

To our knowledge there has been no research to date which has considered the accessibility of PA facilities using specific modes of transport. The research reported in this paper seeks to address this gap in the literature. Previously we have examined the association between area-based income deprivation and the availability of PA facilities within walking and cycling distance [20]. In general, we found that more affluent areas in Scotland were less well served. However, this offers only a partial view of the socio-spatial distribution of accessibility as it ignores those PA facilities that can be reached by motorised modes of transport. It is possible, for example, that lower levels of accessibility by walking and cycling are offset by higher levels of car accessibility in more affluent areas; and, if this were to be the case, those living in more affluent areas would be more likely to be in a position to take advantage of a higher level of car accessibility given that income is a strong predictor of household car ownership (see, for example [21]). Similarly, the accessibility offered by public transport can be expected to vary spatially and temporally and to depend on factors which relate to both the built environment and the socio-economic characteristics of the catchment population. It is therefore important to take motorised transport into account in order to more fully understand how the accessibility of PA facilities varies by socio-economic status.

The purpose of this paper is to examine how access to PA facilities by car and bus varies by area socio-economic status and across urban and rural areas in mainland Scotland. More specifically, we test the hypotheses that the most affluent areas enjoy the highest levels of accessibility to PA facilities by car and the lowest levels of accessibility by bus. Geographical Information System (GIS) car and bus transport networks were created and combined with spatially referenced population and PA facility datasets to model the distribution of access to PA facilities.

## Methods

### Study Area and Population Data

Although Scotland has around one third of the land mass it has less than 10% of the population of Great Britain with just over 5 million inhabitants. Most of the population live in and around the four largest cities of Glasgow, Edinburgh, Aberdeen and Dundee. Large areas of the rest of the country are sparsely populated.

The area used in this study comprised mainland Scotland, which excludes the Orkney and Shetland Islands and the Western Isles. This area was broken down into 6,412 datazones which are the smallest geographical unit routinely used for population statistics in Scotland [22]. For each datazone (DZ), three publicly available area-level variables were used: estimates of the resident population from the 2001 Census [23], the 2006 Scottish Index of Multiple Deprivation (SIMD) income domain scores [24] and the Scottish Executive six-fold Urban Rural Classification (URC) [25].

The 2001 Census was considered to be the most reliable source of population data. Although the updates to the 2001 Census population data have been published each year using data on births and deaths these updates are not reliable for all small areas across Scotland because of the effects of inward and outward migration. The population of DZs ranged from 477 to 2815 with a mean of 779.

The Scottish Government publishes relative deprivation scores for each DZ in seven domains, including current income, employment and crime, as well as an overall index of multiple deprivation [24]. We chose to use the current income domain score because the overall index reflects information on access to services which would be expected to be collinear with the dependent variable in our study. The current income domain score is calculated by dividing the number of adults and their dependents in receipt of financial welfare benefits within a DZ by the total population [24]. We then ranked DZs by their current income domain score in descending order and grouped them into quintiles (Q1 = most affluent, Q5 = most deprived).

The URC system is based on settlement size and, for smaller settlements, drive time to settlements of more than 10,000 or more people. The classification system ranges from large urban areas to remote rural areas [25].

Table 1 shows the distribution of the study area population by URC and the income domain deprivation quintile. It can be seen that large urban areas have a disproportionately high number of individuals living in the most income deprived areas. This is also true of individuals living in deprivation category 3 in remote small towns, deprivation category 2 in accessible rural areas and deprivation categories 2 and 3 in remote rural areas. The percentage of the population living in the most deprived areas in small towns and rural areas is disproportionately low.

**Table 1:** Number of datazones, population of mainland Scotland (2001) and percentage of total population by income deprivation and urban/rural classification.

	Urban Rural Classification																	
Deprivation Quintile	Large urban areas			Other urban areas			Accessible small towns			Remote small towns			Accessible rural areas			Remote rural areas		
	DZs	Pop’n	%	DZs	Pop’n	%	DZs	Pop’n	%	DZs	Pop’n	%	DZs	Pop’n	%	DZs	Pop’n	%
1 (most affluent)	511	413,901	20.86	403	306,154	19.76	138	105,503	22.86	19	14,311	8.51	175	135,752	24.19	46	34,049	12.53
2	371	303,625	15.30	301	231,331	14.93	103	80,749	17.50	41	31,254	18.58	298	223,008	39.74	160	114,502	42.15
3 (middling)	324	257,896	12.99	414	321,459	20.75	148	113,127	24.52	76	58,053	34.50	179	131,867	23.50	126	93,656	34.48
4	465	374,708	18.88	510	389,499	25.14	141	106,113	23.00	59	43,592	25.91	75	58,924	10.50	28	20,585	7.58
5 (most deprived)	783	634,490	31.97	391	300,958	19.42	71	55,926	12.12	30	21,045	12.51	15	11,683	2.08	11	8,850	3.26
	2,454	1,984,620	100	2,019	1,549,401	100	601	461,418	100	225	168,255	100	742	561,234	100	371	271,642	100

Over 60% of households living in the most deprived quintile of DZs and in large urban areas do not own a car or van (Table 2) [26]. This percentage decreases with increasing affluence. Nonetheless even in the most affluent parts of large urban areas just over 17% of households do not own a car. Although car ownership increases with increasing rurality and remoteness around 40% of households in the most deprived DZs in rural areas do not own a car. This figure decreases to around 8% of households in the most affluent DZs.

**Table 2:** Total number of households and percentage which do not own a car (2001) by income deprivation and urban/rural classification.

	Urban Rural Classification											
	Large urban areas		Other urban areas		Accessible small towns		Remote smalltowns		Accessible rural areas		Remote rural areas	
Deprivation Quintile	H/holds	% without a car	H/holds	% without a car	H/holds	% without a car	H/holds	% without a car	H/holds	% without a car	H/holds	% without a car
1 (most affluent)	171,084	17.38	114,575	9.29	39,824	8.78	6,498	12.70	46,919	8.34	12,942	8.72
2	133,497	28.67	96,037	20.78	34,646	18.30	13,489	21.37	89,863	13.13	42,954	14.19
3 (middling)	129,587	40.82	128,312	30.89	45,155	29.08	25,468	30.66	55,780	21.71	48,264	20.84
4	168,625	48.82	178,446	39.82	45,327	37.61	21,696	38.74	26,269	32.05	16,072	31.36
5 (most deprived)	292,168	63.89	143,476	51.85	27,866	45.94	9,319	52.52	6,337	43.96	4,298	42.76

### Recreational Physical Activity Facilities

A data set of all formal recreational PA facilities in Scotland, such as sports centres, football pitches and tennis courts, at June 2007 and their Ordnance Survey grid references [27] was obtained from sportscotland, the national agency for sport in Scotland [28]. As the data set did not contain details of opportunities to undertake physical activity in informal settings (e.g. parks and footpaths) the accessibility of opportunities of this kind was not examined in this study. A full description of the compilation and characteristics of this data set is given by Lamb *et al*. [16]. The original data set contained 14,728 PA facilities which were grouped into 63 different classifications including both permanent facilities, such as football pitches, and other facilities used intermittently for PA, such as school and church halls designated as ‘occasional sports halls’. In total, 359 facilities were omitted from the data set prior to the analysis because they were listed as closed or were assumed not to be open to the general public, such as football pitches used by professional teams. It was not uncommon for more than one PA facility to have the same grid references (e.g. swimming pool and weights room) indicating multiple facilities at a single location. Each individual facility was recorded separately in the data set.

Facilities were grouped into three categories of ownership: public, private and other. The ‘public’ category comprised those owned by local authorities, community enterprises, trusts and voluntary bodies; the ‘private’ category comprised those identified as private, club, commercial or hotel facilities; and the ‘other’ category comprised those found within schools and churches, universities and colleges and those within other workplaces.

### Transport Networks

Car and bus networks were created using TransCAD version 5.0 [29]. Consideration was given to the creation of a public transport network which would incorporate bus, rail and other public transport modes such as the underground system in Glasgow. However, it became clear that nearly all facilities which were classed as being accessible by rail or other public transport modes were also deemed accessible by bus using the chosen measure of accessibility (see below) and so it was decided to concentrate solely on the development of a bus network to represent public transport accessibility.

The car and bus networks were based on the Ordnance Survey Mastermap Integrated Transport Network (ITN) Layer covering mainland Scotland [27]. The origins of journeys to PA facilities were taken to be the population weighted rather than the geometric centroids of DZs in order to guard against bias in the calculation of travel times [30]. The population weighted centroids of DZs and the locations of the PA facilities in the sportscotland dataset were imported into TransCAD, and dummy links were added to connect these point features to the nearest node on the ITN layer. Figure S1 illustrates part of the resulting GIS model for the city of Edinburgh.

The car network was designed to represent uncongested road conditions. Road links were classified by type (that is, motorway, A road, B road, minor road and local street), nature (that is, dual- or single-carriageway) and permitted direction (that is, one- or two-way) using attributes supplied with the ITN layer data. Roads were further classified into urban and rural areas by overlaying the road network with Scottish urban footprint boundary data. Uninterrupted flow speeds were then assigned to each category of road based on the expected speed limit of the road; for example, the speed assigned to an A class single carriageway was 48 km/h in urban areas and 97 km/h in rural areas. Time penalties were applied to left and right turning traffic movements at junctions to reflect the delay experienced by vehicles negotiating the geometry of the junction in accordance with values estimated by McDonald et al. [31].

The bus route system was created using timetable information obtained from the National Public Transport Data Repository [32]. This dataset contained details of all bus stop locations and scheduled bus journeys in mainland Scotland during a selected week in October 2007. Nodes were created on road links to represent the positions of bus stops in the road network. A macro, created using TransCAD’s development platform, was used to identify unique routes, i.e. sets of scheduled journeys which followed a common sequence of bus stops. A total of 12,371 unique routes were identified and each route had one or more scheduled bus journeys associated with it. Routes were then mapped in TransCAD by taking the shortest path by distance on the road network between consecutive pairs of bus stops.

Two potential sources of error were identified in the development of the bus route system: (1) the incorrect ordering of stops in the original dataset and (2) mistakes or major inaccuracies in bus stop coordinates. A verification process was developed to identify and correct those errors that would have a significant effect on in-vehicle journey times or walk access to and from bus services. This involved examining the characteristics of the path taken by each route using a specially-written macro which identified routes that used a road link more than once. This characteristic was found to be indicative of one or the other type of error. Routes identified using this process were examined visually and, for each route, a decision was taken either to accept the mapped route or to modify the input data and recreate the route. The first type of error was addressed by manually editing the original data. Google Maps satellite images [33] showing bus stop locations were used to rectify the second type of error by identifying the correct location of the bus stop and editing the position of the stop in TransCAD. Errors were often found to be replicated across routes which reduced the time spent verifying the bus route system. A total of 430 routes were found to contain errors and were amended using this procedure.

Bus routes operating in four time periods – Wednesday (10∶00 am to 4∶00 pm, 7∶00 pm to 9∶00 pm and 9∶00 pm to 10∶00 pm) and Sunday (10∶00 am to 4∶00 pm) were selected to represent bus services on weekday inter-peak, weekday early evening, weekday late evening and weekend periods respectively. For each of these periods a bus network was created which incorporated the surrounding road network for access, egress and interchange trip stages. The route headway (i.e. the duration between scheduled services) within each selected time period was then calculated.

### Measurement of Accessibility

A variety of techniques to measure accessibility have been proposed (for reviews of the literature, see [34]–[36]). Simple measures of accessibility, such as travel time to the nearest facility, were rejected because of the diversity of PA facilities contained in the sportscotland dataset and the unrealistic nature of the underlying behavioural assumption – i.e. the assumption that individuals choose the nearest PA facility. Other approaches can be classified as location-based, person-based and utility-based methods. Although offering distinct advantages, the latter two methods place a higher information burden on the analyst which cannot always be met within the resource constraints of a project. As a result, we employed a location-based measure of accessibility which reflects the cumulative opportunity for individuals to reach PA facilities and takes the form:

where *A_i_* is the accessibility at origin *i* (taken as the place of residence) to PA facilities at destinations *j* = 1 to *n*, a_j_ is a count of the number of PA facilities at location *j*, t_ij_ is the network travel time between i and j and *f(t_ij_)* is a function which is equal to 1 if facilities at *j* can be reached within a specified travel time threshold and 0 otherwise [35]. In other words, accessibility was taken to be the sum of PA facilities which can be reached from a given origin within a specified travel time. In order to explore how accessibility varied by travel time threshold, we employed thresholds of 10, 20 and 30 minutes in this study.

### Analysis

A matrix of car and bus travel times between population weighted DZ centroids and PA facilities was determined, based on the assumption that travellers would select the shortest path by travel time. For the bus networks, the maximum number of transfers between bus services was limited to two and the maximum access and egress walk times were set at 30 minutes. Walk times and bus stop waiting times were unweighted with respect to in-vehicle travel time. No account was taken of the potential impact of topography or delays experienced at junctions or in crossing roads on walk times. Bus stop waiting times were estimated to be equal to half of the headway up to a maximum of five minutes. This was based on the assumption that passengers would have knowledge of bus timetables and would therefore schedule their travel to avoid excessive waiting times.

This matrix was used to compute the number of PA facilities (in total and broken down by ownership categories) which could be accessed within each travel time threshold from the centroid of each DZ. Accessibility by bus was determined for weekday inter-peak, weekday evening and Sunday afternoon time periods. To account for the anticipated reduction in bus services later in the evening, two criteria were used to determine whether a facility was accessible in the weekday evening period. Firstly, the outward travel time to the facility in the early evening period was required to be less than the specified travel time threshold. Secondly, it had to be possible to make the return journey home in the late evening period in less than 30 minutes.

The median number of accessible facilities within each Income SIMD quintile and each URC category were then calculated. To take into account the effect of population density, we also adjusted the number of accessible facilities for the population of each DZ, as estimated from the 2001 Census, by calculating the median number of accessible facilities per 1,000 individuals. One limitation of our approach is that full account is not taken of the potential demand for PA facilities from the catchment area served by each facility [36]. This is an important consideration in situations where demand is likely to affect the quality of service offered by facilities – for example, high levels of demand could result in overcrowding of facilities at certain times or difficulty in booking preferred time slots. Methods to take this effect into account by adjusting accessibility for potential demand in the catchment areas of facilities have been proposed in the literature [37]–[39]. However, we decided against adjusting for population demand in this way because information relating to the capacity of facilities was not available. We also took the view that the potential demand arising from catchment areas defined using a travel time threshold (as is the case with cumulative opportunities measures) was likely to result in the over-adjustment of accessibility.

Multi-level regression models, which take into account the nesting of DZs within local authorities, were used to identify evidence of statistically significant associations between the number of accessible PA facilities and Income SIMD. An offset of the logarithm of the DZ population was used in the models. Initially Poisson regression models were fitted and the models checked for overdispersion in the outcome variable. Overdispersion occurs when the ratio of the mean to the variance is much greater than one. If overdispersion (taken, in our analysis, to occur when the ratio of mean to variance was greater than two) was found, then a negative binomial model – which has an additional parameter to adjust for overdispersion – was used.

A significant interaction was found between URC and Income SIMD. Separate models were therefore fitted for urban areas (consisting of URC categories 1 and 2: large urban areas and other urban areas), small town areas (URC categories 3 and 4: accessible small towns and remote small towns) and rural areas (URC categories 5 and 6: accessible rural areas and remote rural areas).

Moran's I permutation test was carried out to test the null hypothesis that there is no spatial autocorrelation between DZs sharing a common border. Where statistically significant spatial autocorrelation was detected, a spatial weighting variable based on that proposed by Lee and Neocleous [40] was included in the regression models. This variable is dependent on the response of adjacent DZs and is expressed as:

where *y_i_* is the number of PA facilities accessible from DZ *i* and *x_i_* is the total population in DZ *i* which was used as the offset in the modelling. Due to the presence of zero observations in some response variables, a correction factor of 0.5 was added to the variable. The weights are specified so that *ω_ki_ = *1 if areas (*k, i*) share a common border and zero otherwise. *ω_kk_ = *0 for all *k*. Although the spatial weighting variable reduced the residual spatial autocorrelation, the correlation remained statistically significant in some cases. Therefore, a more conservative 99% level of significance was adopted in the analysis rather than the more conventional 95% level. All statistical analysis was conducted using R version 2.11.1 [41].

## Results

### Descriptive Statistics

Table 3 shows the unadjusted and population adjusted median, minimum and maximum number of PA facilities accessible within 10, 20 and 30 minute travel time thresholds by car from population weighted centroids of DZs. Overall, the median number of accessible facilities increased with increasing deprivation from the second most affluent quintile (Q2) to the most deprived quintile (Q5) of DZs for each travel time threshold examined. However, the level of accessibility afforded to DZs in the most affluent quintile (Q1) was higher than that for all but the most deprived quintile (Q5) for the 10 minute threshold and was higher than that for the second and third most affluent quintiles (Q2 and Q3) for the 20 and 30 minute thresholds. Overall, the median number of accessible facilities decreased with decreasing settlement size and increasing remoteness for all time thresholds considered.

**Table 3:** Unadjusted and population adjusted§ median and range of number of recreational physical activity facilities within 10, 20 and 30 minutes travel time by car by income deprivation quintile and urban/rural classification.

	10 minutes		20 minutes		30 minutes	
	Unadjusted	Adjusted	Unadjusted	Adjusted	Unadjusted	Adjusted
**Deprivation Quintile**						
1 (most affluent)	271 (0, 1130)	350.6 (0, 2063.7)	1009 (0, 2342)	1354.2 (0, 4351.3)	2286.5 (12, 3837)	2636.9 (17.1, 6702.2)
2	142 (0, 1182)	189.4 (0, 2155.3)	538 (0, 2339)	714.9 (0, 4206.4)	1279.5 (1, 4105)	1679.1 (1.8, 7437)
3 (middling)	164 (0, 1203)	222.0 (0, 2237.7)	589 (0, 2344)	820.7 (0, 4234)	1533 (1, 4168)	2002.6 (1.1, 7281.8)
4	243.5 (3, 1211)	332.5 (4.4, 1989.7)	1073 (3, 2370)	1411.7 (5.9, 4677.6)	2325 (4, 4223)	2910.8 (6.8, 7810.9)
5 (most deprived)	434 (7, 1196)	563.1 (7.1, 2208.6)	1666 (21, 2382)	2007.6 (21.6, 4378.7)	2774 (21, 4140)	3201.8 (24.6, 7039.5)
**Urban/rural classification**						
1 (large urban)	763 (163, 1211)	911.2 (199.2, 2237.7)	1896 (452, 2382)	2273.0 (397.9, 4677.6)	2831 (702, 350.4)	3310.8 (490.2, 6611.9)
2 (other urban)	172 (24, 695)	227.9 (25.2, 1259.3)	733 (54, 2221)	981.4 (53.5, 4066.4)	2092 (60, 4233)	2661.0 (63.4, 7810.9)
3 (accessible small towns)	103 (13, 992)	137.4 (14.3, 1467.4)	547 (32, 2325)	731.4 (35.9, 4351.3)	1671 (72, 4082)	2135.4 (79.1, 6425.1)
4 (remote small towns)	30 (14, 56)	38.0 (15.3, 97.6)	76 (20, 351)	98.4 (21.6, 530.7)	177 (21, 965)	246.1 (22.3, 1448.3)
5 (accessible rural)	54 (0, 927)	78.0 (0, 1363.2)	330.5 (6, 2282)	442.2 (8.8, 4023.2)	747 (53, 3961)	1095.6 (57.3, 7057.6)
6 (remote rural)	14 (0, 92)	18.8 (0, 154.9)	41 (0, 483)	56.1 (0, 743.5)	94 (1, 1626)	130.3 (1.1, 2552.6)

§ The number of facilities per 1000 individuals.

Table 4 and Table 5 show the variation in accessibility by bus during the weekday inter-peak and weekday evening periods respectively. As expected, the median number of PA facilities accessible was considerably lower by bus than by car and accessibility by bus was higher during the weekday inter-peak time period than in the weekday evening period. The median number of accessible facilities increased with increasing deprivation from Q2 to Q5 for the 20 and 30 minute thresholds and from Q1 to Q4 for the 10 minute threshold. The accessibility of facilities from the most affluent quintile (Q1) was higher than Q2 for the 20 and 30 minute thresholds and also Q3 for the 30 minute threshold (weekday interpeak only).

**Table 4:** Unadjusted and population adjusted§ median and range of number of recreational physical activity facilities within 10, 20 and 30 minutes travel time by bus during the weekday inter-peak period by income deprivation quintile and urban/rural classification.

	10 minutes		20 minutes		30 minutes	
	Unadjusted	Adjusted	Unadjusted	Adjusted	Unadjusted	Adjusted
**Deprivation Quintile**						
1 (most affluent)	1 (0, 28)	1.3 (0, 32.9)	19 (0, 543)	25.1 (0, 731.1)	91 (0, 1111)	117.6 (0, 2047.8)
2	1 (0, 28)	1.7 (0, 37.6)	18 (0, 632)	23.3 (0, 971.6)	71 (0, 1256)	94.9 (0, 1859.1)
3 (middling)	2 (0, 26)	2.7 (0, 34.9)	22 (0, 550)	29.9 (0, 775.7)	83 (0, 1183)	113.6 (0, 1924.2)
4	3 (0, 24)	3.4 (0, 37.2)	34 (0, 517)	44.2 (0, 945.2)	125.5 (0, 1097)	164.0 (0, 2005.5)
5 (most deprived)	2 (0, 22)	3.1 (0, 27.2)	44 (0, 378)	55.7 (0, 634.7)	200 (8, 894)	249.7 (9.7, 1683.6)
**Urban/rural classification**						
1 (large urban)	3 (0, 28)	3.8 (0, 37.6)	64 (0, 632)	80.8 (0, 971.6)	297 (11, 1256)	374.4 (18.9, 2047.8)
2 (other urban)	2 (0, 20)	2.0 (0, 31.9)	25 (0, 177)	32.8 (0, 319.4)	88 (2, 479)	115.6 (2.9, 845.7)
3 (accessible small towns)	2 (0, 22)	2.4 (0, 30.8)	19 (0, 133)	24.5 (0, 231.8)	68 (7, 558)	91.7 (9.2, 793.1)
4 (remote small towns)	2 (0, 20)	3.1 (0, 23.8)	13 (0, 38)	17.4 (0, 67)	22 (3, 78)	32.4 (3.1, 114.1)
5 (accessible rural)	1 (0, 12)	1.4 (0, 17.8)	7 (0, 105)	10.1 (0, 157.8)	33 (0, 466)	44.4 (0, 646.5)
6 (remote rural)	0 (0, 12)	0 (0, 17.5)	2 (0, 46)	3.0 (0, 71.3)	7 (0, 105)	8.8 (0, 159.7)

§ The number of facilities per 1000 individuals.

**Table 5:** Unadjusted and population adjusted§ median and range of number of recreational physical activity facilities within 10, 20 and 30 minutes travel time by bus during the weekday evening period by income deprivation quintile and urban/rural classification.

	10 minutes		20 minutes		30 minutes	
	Unadjusted	Adjusted	Unadjusted	Adjusted	Unadjusted	Adjusted
**Deprivation Quintile**						
1 (most affluent)	1 (0, 24)	1.3 (0, 32.9)	14 (0, 525)	17.8 (0, 659.4)	53 (0, 994)	70.1 (0, 1665.3)
2	1 (0, 24)	1.7 (0, 33.4)	13 (0, 605)	16.5 (0, 867.4)	45 (0, 1174)	57.5 (0, 1772.2)
3 (middling)	2 (0, 22)	2.7 (0, 32.0)	18 (0, 513)	24.1 (0, 723.6)	55 (0, 1087)	72.7 (0, 1626.4)
4	3 (0, 26)	3.3 (0, 44.8)	25 (0, 527)	33.6 (0, 963.4)	82 (0, 997)	105.4 (0, 1822.7)
5 (most deprived)	2 (0, 23)	3.1 (0, 27.9)	34 (0, 316)	42.6 (0, 457.6)	115 (1, 736)	150.0 (1.3, 1293.8)
**Urban/rural classification**						
1 (large urban)	3 (0, 26)	3.6 (0, 44.8)	50 (0, 605)	64.2 (0, 958.2)	175 (2, 1174)	223.9 (3.2, 1812.7)
2 (other urban)	1 (0, 20)	1.9 (0, 31.8)	18 (0, 174)	23.9 (0, 316.9)	59 (0, 393)	77.8 (0, 715.9)
3 (accessible small towns)	2 (0, 22)	2.4 (0, 30.5)	15 (0, 127)	19.5 (0, 221.6)	38 (1, 372)	50.7 (1.9, 539.9)
4 (remote small towns)	2 (0, 20)	3.0 (0, 23.81)	12 (0, 38)	16.5 (0, 65.1)	16 (1, 73)	23.5 (1.0, 97.7)
5 (accessible rural)	1 (0, 12)	1.4 (0, 17.8)	4 (0, 85)	4.8 (0, 137.2)	6 (0, 268)	8.6 (0, 509.5)
6 (remote rural)	0 (0, 12)	0 (0, 17.4)	1 (0, 46)	1.9 (0, 71.7)	2 (0, 99)	3.1 (0, 154.2)

§ The number of facilities per 1000 individuals.

Our results showed a large variation in the accessibility of PA facilities by car and bus within each deprivation quintile and urban rural category. Whilst some DZs in each category were very well-catered for in terms of PA facilities, it was also clear that there were DZs with only a few accessible PA facilities. This is particularly the case for DZs in all income deprivation and urban/rural class outside large urban areas by bus and for remote areas and Q2 and Q3 by car.

### Regression Models

**Figure 1:**
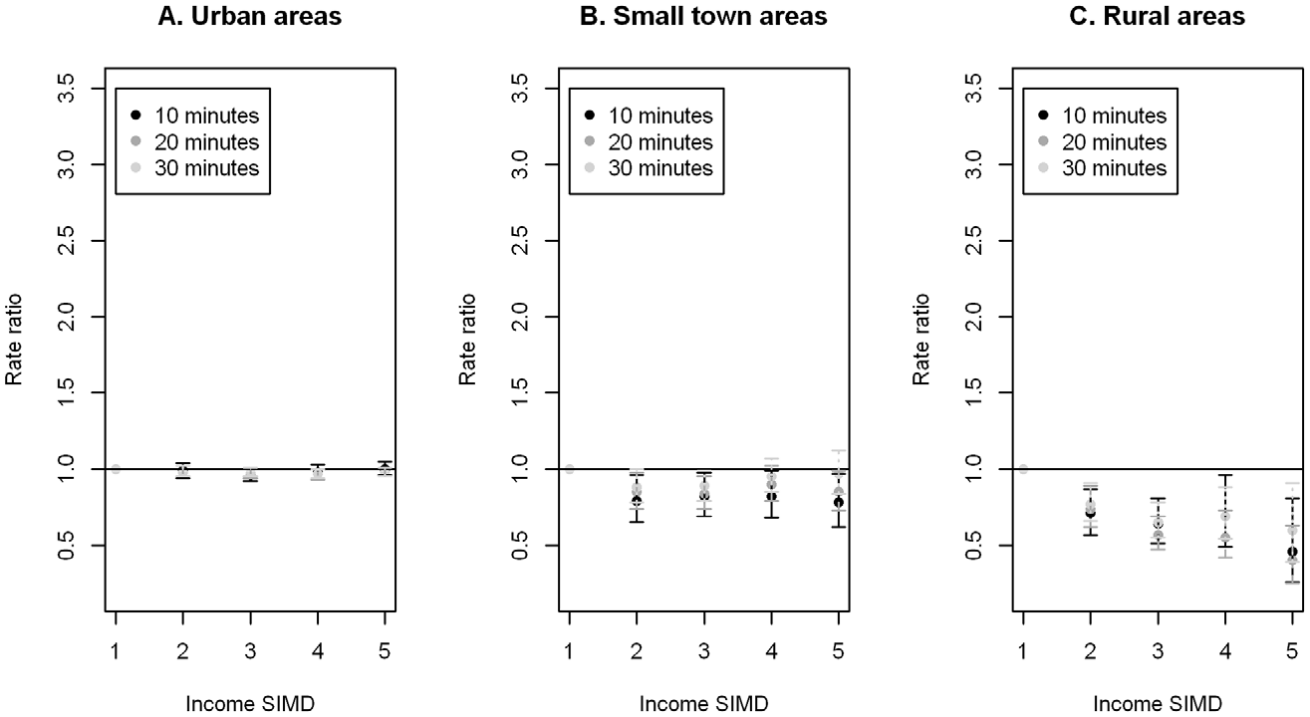
Car access to PA facilities by income deprivation. Adjusted rate ratios and 99% confidence intervals of PA facilities which are accessible by car within a travel time of 10, 20 and 30 minutes of A. urban, B. small town and C. rural areas by income deprivation, 1 = most affluent (reference category) to 5 = most deprived.

**Figure 2:**
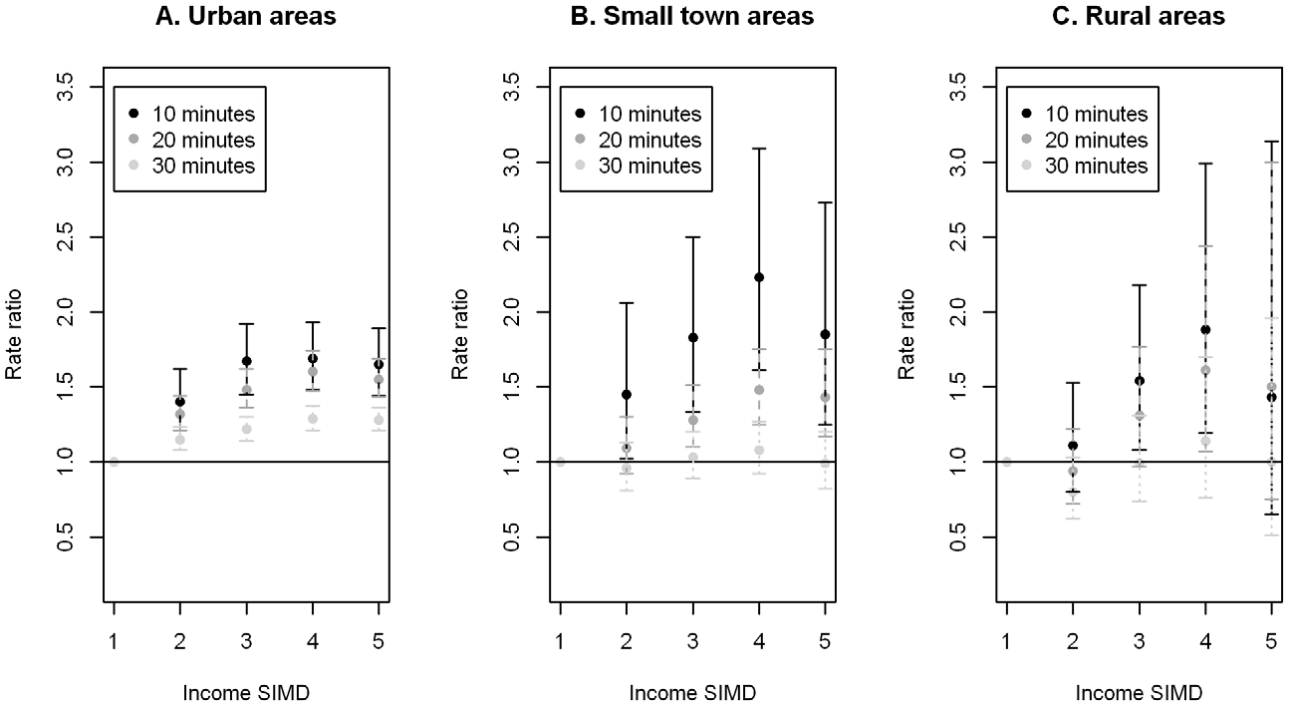
Bus access to PA facilities by income deprivation. Adjusted rate ratios and 99% confidence intervals of PA facilities which are accessible by bus within a travel time of 10, 20 and 30 minutes of A. urban, B. small town and C. rural areas by income deprivation, 1 = most affluent (reference category) to 5 = most deprived.

Whilst the patterns for car and bus accessibility appear similar from the descriptive statistics, marked differences are shown in accessibility by these two modes of transport when looking at urban, small town and rural areas separately in the modelling which adjusted for population and random local authority effects (as well as spatial correlation, where appropriate). Figure 1 and Figure 2 show the adjusted rate ratios (RRs) with 99% confidence intervals (CIs) for PA facilities accessible by car and bus (weekday inter-peak) within a travel time of 10, 20 and 30 minutes. Rate ratios are the ratio of the rate of an event (i.e. the number of facilities per individual) for one group (for example, income deprivation quintile 2) relative to that for another group (income deprivation quintile 1). RRs greater than 1 indicate higher accessibility of facilities than the comparator category, which in this case was the most affluent quintile (Q1). (Table S1 and Table S2 present detailed model parameter estimates for car and bus respectively).

In urban areas, no statistically significant differences in accessibility by car were found between Q1 and the other deprivation quintiles. For small towns, Q3 had a significantly lower RR than Q1 within 30 a minute travel time by car, although no clear trend is evident between the level of deprivation and the accessibility of facilities. Q2 to Q5 had significantly lower RRs than Q1 for the 10 minute threshold and Q2, Q3 and Q5 had significantly lower RRs than Q1 for the 20 minute threshold. In rural areas, Q2 to Q5 all had significantly lower RRs than Q1 at the 10, 20 and 30 minute car travel time thresholds, and the estimated RRs for Q5 were the lowest in each case.

For accessibility by bus in urban areas, Q2 to Q5 had significantly higher RRs than Q1, with a trend of increasing RR with increasing deprivation from Q1 to Q4. This was also the case for small towns for the 10 and 20 minute thresholds (with the exception that Q2 was not significantly different from Q1 using the 20 minute threshold). However, there were no significant differences between the RRs at the 30 minute threshold and no clear trend was evident. For rural areas, Q3 and Q4 were significantly higher than Q1 using the 10 minute threshold, but only Q4 was significantly higher than Q1 for the 20 minute threshold. No significant differences were detected at the 30 minute threshold.

Separate models were constructed for subcategories of facilities in public and private ownership the results of which are presented in Table 6 and Table 7 respectively. In general, these models produced similar results to those obtained from the models discussed above. One notable difference was observed between the accessibility of public and private facilities by bus in urban areas for the 10 minute threshold (but not for the 20 or 30 minute thresholds). For public facilities, Q2 to Q5 were found to have significantly higher RRs than Q1 with a trend of increasing RR with deprivation. In contrast, for private facilities, Q3 had a significantly higher RR than Q1 and Q5 had a significantly lower RR than Q1–Q3. Also, in rural areas, using the 10 minute bus threshold, we found that the RRs of public facilities were significantly higher in Q3 to Q5 than Q1, whereas no significant differences were detected in the RRs of private facilities.

**Table 6:** Car access to public and private PA facilities by income deprivation.

Deprivation Quintile	10 minutes				20 minutes				30 minutes			
	Public		Private		Public		Private		Public		Private	
(a) Urban	RR	99% C.I.	RR	99% C.I.	RR	99% C.I.	RR	99% C.I.	RR	99% C.I.	RR	99% C.I.
1	1		1		1		1		1		1	
2	1.00	(0.95, 1.04)	1.00	(0.95, 1.05)	1.01	(0.98, 1.05)	1.02	(0.98, 1.05)	1.00	(0.96, 1.03)	1.01	(0.98, 1.04)
3	0.97	(0.92, 1.01)	0.97	(0.93, 1.03)	1.00	(0.97, 1.04)	1.00	(0.96, 1.04)	0.99	(0.96, 1.02)	1.00	(0.97, 1.03)
4	0.99	(0.95, 1.04)	0.99	(0.95, 1.04)	0.97	(0.94, 1.01)	0.96	(0.93, 0.997)	0.97	(0.95, 1.00)	0.98	(0.95, 1.01)
5	1.01	(0.97, 1.05)	1.01	(0.96, 1.06)	0.98	(0.95, 1.02)	0.97	(0.94, 1.01)	0.98	(0.95, 1.00)	0.98	(0.95, 1.01)
(b) Small Town												
1	1		1		1		1		1		1	
2	0.78	(0.64, 0.95)	0.83	(0.70, 0.99)	0.82	(0.71, 0.96)	0.81	(0.69, 0.95)	0.88	(0.77, 1.00)	0.86	(0.75, 0.99)
3	0.81	(0.68, 0.97)	0.88	(0.75, 1.03)	0.83	(0.72, 0.95)	0.83	(0.72, 0.95)	0.86	(0.76, 0.97)	0.86	(0.76, 0.97)
4	0.82	(0.68, 0.996)	0.84	(0.71, 0.995)	0.89	(0.77, 1.03)	0.86	(0.74, 1.00)	0.93	(0.82, 1.06)	0.91	(0.80, 1.04)
5	0.79	(0.63, 0.99)	0.84	(0.68, 1.02)	0.82	(0.69, 0.98)	0.80	(0.67, 0.95)	0.90	(0.77, 1.05)	0.88	(0.76, 1.03)
(c) Rural												
1	1		1		1		1		1		1	
2	0.71	(0.56, 0.89)	0.77	(0.62, 0.95)	0.70	(0.58, 0.85)	0.70	(0.59, 0.85)	0.73	(0.61, 0.88)	0.74	(0.62, 0.89)
3	0.68	(0.53, 0.88)	0.74	(0.58, 0.93)	0.55	(0.44, 0.67)	0.58	(0.47, 0.71)	0.56	(0.46, 0.69)	0.58	(0.47, 0.70)
4	0.77	(0.54, 1.10)	0.77	(0.56, 1.07)	0.53	(0.40, 0.72)	0.54	(0.40, 0.72)	0.56	(0.42, 0.75)	0.57	(0.43, 0.76)
5	0.65	(0.36, 1.19)	0.49	(0.28, 0.85)	0.43	(0.26, 0.71)	0.36	(0.22, 0.59)	0.48	(0.30, 0.79)	0.46	(0.29, 0.74)

Adjusted rate ratios and 99% confidence intervals for (a) urban, (b) small town and (c) rural areas.

**Table 7:** Bus access to public and private PA facilities by income deprivation.

Deprivation Quintile	10 minutes				20 minutes				30 minutes			
	Public		Private		Public		Private		Public		Private	
(a) Urban	RR	99% C.I.	RR	99% C.I.	RR	99% C.I.	RR	99% C.I.	RR	99% C.I.	RR	99% C.I.
1	1		1		1		1		1		1	
2	1.73	(1.34, 2.23)	1.23	(0.99, 1.53)	1.42	(1.29, 1.57)	1.27	(1.14, 1.42)	1.18	(1.10, 1.26)	1.13	(1.05, 1.21)
3	2.04	(1.59, 2.61)	1.29	(1.04, 1.61)	1.60	(1.45, 1.76)	1.35	(1.21, 1.51)	1.25	(1.17, 1.33)	1.19	(1.11, 1.27)
4	2.30	(1.82, 2.91)	0.99	(0.79, 1.24)	1.71	(1.56, 1.88)	1.40	(1.26, 1.55)	1.34	(1.26, 1.42)	1.26	(1.18, 1.34)
5	2.46	(1.95, 3.11)	0.69	(0.55, 0.88)	1.64	(1.49, 1.80)	1.26	(1.14, 1.40)	1.32	(1.24, 1.41)	1.23	(1.15, 1.32)
(b) Small Town												
1	1		1		1		1		1		1	
2	1.55	(0.93, 2.59)	2.14	(1.15, 3.98)	1.03	(0.82, 1.29)	1.27	(0.99, 1.62)	0.95	(0.80, 1.14)	1.03	(0.85, 1.23)
3	1.65	(1.04, 2.62)	2.43	(1.37, 4.31)	1.20	(0.98, 1.47)	1.52	(1.21, 1.90)	1.03	(0.88, 1.21)	1.16	(0.98, 1.38)
4	2.44	(1.53, 3.91)	2.34	(1.29, 4.24)	1.46	(1.18, 1.80)	1.53	(1.21, 1.95)	1.11	(0.94, 1.32)	1.14	(0.96, 1.36)
5	1.95	(1.11, 3.43)	2.09	(1.04, 4.17)	1.40	(1.09, 1.81)	1.48	(1.11, 1.97)	1.01	(0.82, 1.24)	1.09	(0.88, 1.36)
(c) Rural												
1	1		1		1		1		1		1	
2	1.39	(0.89, 2.16)	0.93	(0.57, 1.52)	1.03	(0.75, 1.42)	0.97	(0.72, 1.29)	0.86	(0.65, 1.14)	0.83	(0.65, 1.06)
3	1.97	(1.26, 3.09)	1.10	(0.66, 1.82)	1.49	(1.05, 2.12)	1.28	(0.93, 1.76)	1.07	(0.79, 1.47)	1.05	(0.80, 1.39)
4	3.15	(1.90, 5.22)	1.02	(0.53, 1.97)	1.85	(1.15, 2.98)	1.31	(0.85, 2.01)	1.34	(0.87, 2.07)	1.10	(0.75, 1.62)
5	2.15	(1.14, 5.50)	0.29	(0.05, 1.83)	1.94	(0.89, 4.22)	1.02	(0.49, 2.13)	1.41	(0.68, 2.91)	0.89	(0.47, 1.71)

Adjusted rate ratios and 99% confidence intervals for (a) urban, (b) small town and (c) rural areas.

## Discussion and Conclusions

In this study we found that in absolute terms, the accessibility by car of recreational PA facilities in Scotland greatly exceeded that by bus, and that this difference was more pronounced for lower travel time thresholds where access and egress times constituted a greater proportion of total bus travel time. We also found that remote areas and, to a lesser extent, accessible rural areas had fewer opportunities to access PA facilities than urban areas and accessible small towns.

Our regression models showed that accessibility by car was significantly (*p*<0.01) higher for the most affluent quintile of area-based income deprivation than for most other quintiles in small towns and all other quintiles in rural areas, but this was not the case in urban areas. In contrast, access to PA facilities by bus for the most affluent quintile was significantly lower than that for other quintiles in urban areas and small towns, but not in rural areas. With the exception of access by car in urban areas and bus in rural areas, these results are consistent with the twin hypotheses that the most affluent areas enjoy the best access to PA facilities by car but the poorest access to these facilities by bus compared to other deprivation quintiles within each particular urban/rural class modelled in this paper.

When we sub-categorised PA facilities by ownership, we did not find any significant difference between private and public PA facility accessibility except for the lowest travel time threshold for bus in urban areas. It might be hypothesised that as a consequence of market forces private facilities are more likely to be located close to more affluent areas and that public policy would seek to redress this imbalance by locating public facilities in more deprived areas. Proximity by distance is closely related to access by bus within 10 minutes since our assessment of total journey time took into account access, egress and bus stop waiting times as well as in-vehicle time. Our results, which take into account the effect of population density, are consistent with this hypothesis to the extent that access to public facilities increased with increasing deprivation, whereas access to private facilities was significantly lower in the most deprived areas than in the more affluent quintiles.

In previous analysis we found that those living in ‘middling’ income areas enjoyed better access to PA facilities within the DZ of residence than those living in either low or high-income areas, after adjusting for population density, urbanicity and local authority [16]. When we then examined the accessibility of facilities within walking and cycling distance we found, in general, that low and middling income areas experienced similar levels of accessibility and that the most affluent areas had poorer access than other areas [20]. We also found that the accessibility gap between the most affluent and other areas became less pronounced with higher travel time thresholds and for cycling in comparison with walking. This shows that any apparent disadvantage experienced by the most deprived areas in comparison with middling income areas can be readily overcome within a relatively short travel time or distance. Furthermore, in this paper our results for car accessibility reveal that a journey of 10 minutes is sufficient to remove any differences in accessibility by area-based income deprivation in urban areas and to provide higher levels of accessibility in small towns and rural areas. This shows the degree to which the relative accessibility of PA facilities is sensitive to small changes in travel time threshold by walking and cycling or to choosing to travel by car for even short journeys.

With reference to the wider literature, to the best of our knowledge the only comparable research was that undertaken in metropolitan Perth, Western Australia which found that low socio-economic status (SES) areas enjoyed better road network access to PA facilities than high SES areas [19]. Clearly, the apparent disparity between that work and our results on car accessibility may be attributable to the different geographical and social contexts of the two study areas. From a methodological perspective, a strength of our study was that it took into account the effect of road type and geometry-related junction delay on travel times, whereas the Australian study was based solely on road network distance. In addition, different approaches were employed in the determination of accessibility. Whereas we used the cumulative opportunities method, a gravity-based approach was used in the Australian study in which the closer a facility to an origin, the greater its contribution to the accessibility index. We found that differences in accessibility by area-based income deprivation increased when we reduced the travel time threshold and, at the lowest threshold examined (10 minutes), the most affluent areas enjoyed as good if not better access by car, a finding which is apparently at odds with the results of the Australian study. However, as discussed in the previous paragraph, if we were to reduce the car travel time threshold to below 10 minutes (or, alternatively, by considering walk/cycle accessibility), we would anticipate a steeper decline in accessibility in the most affluent areas than elsewhere. This suggests that at least some of the difference between the results of the two studies may be attributable to the method used to calculate accessibility, but in the absence of behavioural data to estimate the deterrent effect of travel time (for different modes) in our study area, it is not possible to pursue this question further using currently available data.

It is clear from our analysis that car ownership is an important factor in determining the level of accessibility to PA facilities enjoyed by an individual. We have seen in Table 2 that there is a considerable variation in the proportion of households without access to a car in Scotland and that a large proportion of Scottish households must rely on non-car modes of transport to use PA facilities. Our results for bus accessibility in urban areas and in small towns showed that the most affluent quintile had a lower level of accessibility than other quintiles. This suggests that bus services are weighted in favour of less well-off areas and that this helps to connect the population of these areas with PA facilities. At the same time, the groups which are most disadvantaged according to our results are those without access to a car and living in the most affluent areas or in rural areas.

An obvious extension to our analysis would be to explore the intersection of car ownership with accessibility by income deprivation and URC. Car ownership data is available for households at DZ level in Scotland but the amount of information on the size and structure of households presented with this data is limited. This means that e.g. the number of cars per adult household member is not known. There is also the added difficulty of interpreting the results since individual household members are likely to have unequal access to any cars owned by their household as a result of driving licences held, work commuting and other such constraints. Nonetheless, we performed a tentative analysis in which we calculated a compound accessibility score for each DZ by weighting car and bus population adjusted accessibilities by the proportion of households with and without a car respectively. We then calculated the median compound accessibility for each travel time threshold and income deprivation quintile nested within urban, small town and rural categories. The full results are shown in Table S3. Despite higher car ownership in rural areas, there remained a clear advantage in compound accessibility for urban areas over rural areas. As expected given the positive association between income and car ownership and the gap between car and bus accessibilities, the most affluent quintile enjoyed the highest level of compound accessibility. In urban areas, compound accessibility declined with increasing deprivation for all travel time thresholds. In small towns and rural areas this was also the case for the 10 minute travel time threshold, although no trend was apparent for the 20 and 30 minute thresholds.

This paper is one of the few to have examined the accessibility of PA facilities beyond the level of the neighbourhood and is the first to develop a GIS model of car and bus networks for this purpose. One limitation of the model was that it did not take into account the effect of congestion-related delays on car travel times. Another limitation was that we assumed a constant speed for access to and egress from bus services which did not take into account the effect of the roadway environment on such speeds. Likewise, the effect of bus service frequency on accessibility was not fully accounted for. As described in the methods section, bus stop waiting times were estimated to be a function of the service headway up to a maximum of five minutes, based on the assumption that passengers would have prior knowledge of the timetable and so would schedule their journey accordingly. However, this does not take into account any underutilised time at home or at the PA facility, which would tend to be greater for routes with less frequent services, nor does it capture the effect of constraints placed by (or upon) other activities on journey planning.

With the exception of disaggregating the PA facility data set along public/private ownership lines, we took no account of potential preferences for particular types of facility in our analysis. We know from population health surveys in Great Britain that preferences for different forms of PA vary by age and gender [5]–[6]. It is also reasonable to infer that the attractiveness of different forms of sport/exercise is socially and culturally dependent. We have partly addressed the issue of varying potential preferences for different types of PA facility in a companion paper in which we classified facilities by the intensity of PA offered [42]. With a suitable data set it would also be possible to examine differential access to facilities with specific attributes such as the availability of specialist coaching and reserved sessions for particular demographic groups. It would also be appropriate to widen the scope of the research to include opportunities for PA in informal settings.

The cumulative opportunities accessibility measure used in this research reveals the total number of PA facilities available to an individual within a specified travel time. We examined the accessibility gradient across three travel time thresholds. Our approach did not take into account the distance or time individuals were prepared to travel to reach PA facilities. Recent evidence from Australia revealed that the average network distance travelled to formal recreational facilities was around 5.5km [43]. In future research, therefore, it would be useful to explore how far individuals are prepared to travel to undertake PA in a Scottish context, and how this varies by mode and by sex, gender and other socio-economic characteristics. Furthermore, it is recognised that the value of accessibility above a certain satisfactory and sufficient level depends on the degree, or the diversity, of choice available [44]. This means that, for example, if a community had two accessible swimming pools but no facilities for tennis, the provision of a tennis facility would tend to add more value to the community than the provision of an additional swimming pool. This “value” may arise in satisfying a taste for variety in PA (e.g. using facilities with different attributes, taking part in different forms of PA) or in providing opportunities for novices to try out different forms of sport/exercise. The provision of an appropriate mix of facilities is also important in satisfying different preferences at household or community level as discussed in the preceding paragraph. Alternative formulations of accessibility have been proposed in the literature to capture diversity (see, for example, [45]) and there is clearly scope to extend our research in this direction.

The Scottish Government recognises the link between poor health and deprivation [46] and it views increasing physical activity as an important component of its policy to tackle ill-health [47]–[48]. Furthermore, increasing participation in sport is regarded as an important legacy of the 2012 London Olympic Games [49] and the XX Commonwealth Games in Scotland [50]. The aim of this study was to examine how accessibility to PA facilities by car and bus varies by area deprivation and thereby identify which groups are least well-served by the provision of PA facilities. We have shown that, when considering urban areas and small towns, residents of the most affluent areas without access to a car have the least good access to PA facilities. (Table 2 shows that a non-trivial number of households in the most affluent areas of Scotland do not own a car). This highlights the need for planners to consider all those without access to a car, not simply those living in more deprived areas, when determining the number and location of public PA facilities and in securing commitments towards local PA facilities from developers of new housing schemes. It has particular implications for the construction of affordable housing within larger housing developments on the periphery of existing settlements where high quality public transport is less likely to be commercially viable. This means that households without access to a car would be better off (in terms of access to PA facilities) if they were to live in more deprived areas. Even for low-income households with access to a car and living in the most affluent areas, the proportion of income spent on transportation tends to be particularly high [51]. Part of the solution, therefore, lies in ensuring that public policy encourages investment in local PA facilities where possible. This would also have the effect of integrating and aligning policies on sports participation and reduction in carbon emissions from motorised transport. Where this is not possible, for reasons of finance, low rates of demand, lack of available land and suchlike, consideration should be given to clustering PA facilities in areas of high public transport accessibility. Measures to support cycling which provides relatively inexpensive access to mid-range destinations within a reasonable travel time would also be valuable. The picture in remote and rural areas is different in that there is no clear trend in accessibility of PA facilities with increasing deprivation. Although relevant, the policy measures discussed above are likely to play a less important role because either they are more difficult to implement or are less effective largely as a result of lower population densities. One of the key problems across parts of Scotland is the lack of access to a regular bus service. In these situations, provision of demand responsive transport offering greater flexibility and wider network coverage is an important part of the solution [52].

## Supporting Information

Figure S1 Map of the city of Edinburgh which formed part of the study area. The map illustrates the GIS transport network model and shows the locations of PA facilities (by ownership type - public, private and other) and the road and bus networks (by service frequency).
(DOC)

Table S1 Rate ratio of PA facilities accessible by car within a travel time of 10, 20 and 30 minutes of urban, small town and rural areas by income deprivation.
(DOC)

Table S2 Rate ratio of PA facilities accessible by car within a travel time of 10, 20 and 30 minutes of urban, small town and rural areas by income deprivation.
(DOC)

Table S3 Median compound accessibility by income deprivation and urban/rural classification.
(DOC)
