# Supplementary material for: Access to Recreational Physical Activities by Car and Bus: An Assessment of Socio-Spatial Inequalities in Mainland Scotland
Source: PLoS One. 2013 Feb 7;8(2):e55638. doi: 10.1371/journal.pone.0055638 (PMC3567099; doi:10.1371/journal.pone.0055638)
Supplement: Figure S1 — Map of the city of Edinburgh which formed part of the study area. The map illustrates the GIS transport network model and shows the locations of PA facilities (by ownership type - public, private and other) and the road and bus networks (by service frequency). (DOC) [file pone.0055638.s001.doc]

Figure S1 Map of the city of Edinburgh which formed part of the study area. The map illustrates the GIS transport network model and shows the locations of PA facilities (by ownership type - public, private and other) and the road and bus networks (by service frequency)


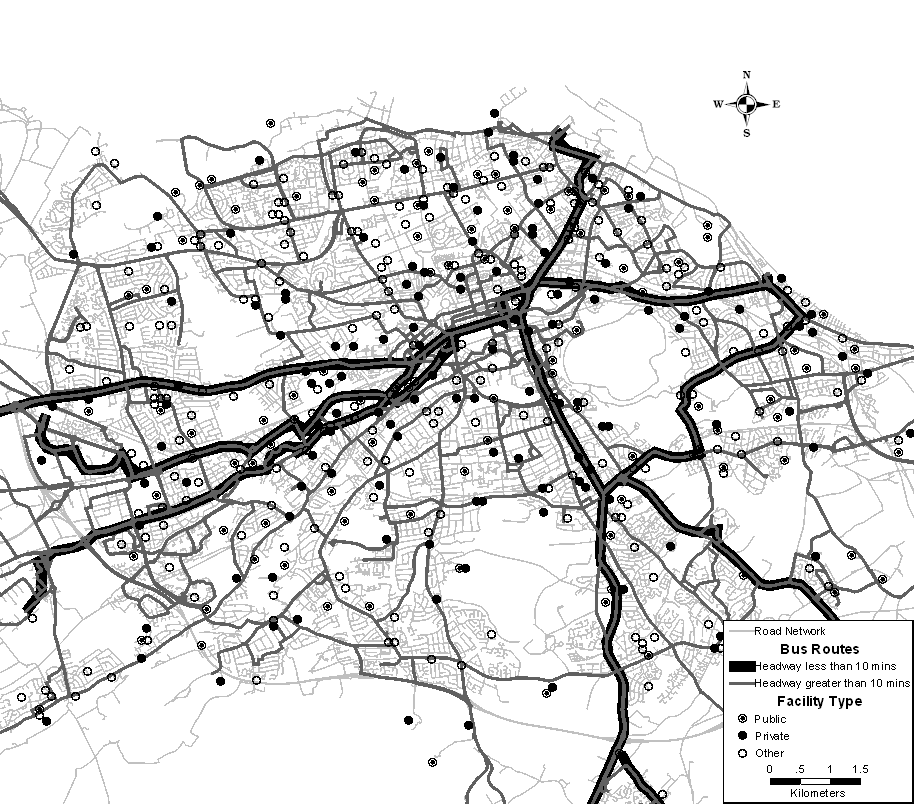


© Crown Copyright/database right 2012. An Ordnance Survey/(Datacentre) supplied service
