# Supplementary material for: Access to Recreational Physical Activities by Car and Bus: An Assessment of Socio-Spatial Inequalities in Mainland Scotland
Source: PLoS One. 2013 Feb 7;8(2):e55638. doi: 10.1371/journal.pone.0055638 (PMC3567099; doi:10.1371/journal.pone.0055638)
Supplement: Table S1 — Rate ratio of PA facilities accessible by car within a travel time of 10, 20 and 30 minutes of urban, small town and rural areas by income deprivation. (DOC) [file pone.0055638.s002.doc]

**Table S1 Rate ratio of PA facilities accessible by car within a travel time of 10, 20 and 30 minutes of urban, small town and rural areas by income deprivation**

**Table S1(a)** PA facilities accessible by car within 10 minutes

|  | **Urban** | **Small town** | **Rural** |
| --- | --- | --- | --- |
| **Deprivation Quintile** | **Coefficient (99% CI)** | **Coefficient (99% CI)** | **Coefficient (99% CI)** |
| Intercept | -1.22 (-1.62, -0.82) | -1.85 (-2.33, -1.36) | -1.42 (-1.92, -0.91) |
| 2 | -0.01 (-0.06, 0.04) | -0.23 (-0.42, -0.04) | -0.35 (-0.56, -0.13) |
| 3 (middling) | -0.04 (-0.09, 0.01) | -0.19 (-0.37, -0.02) | -0.45 (-0.68, -0.21) |
| 4 | -0.02 (-0.06, 0.03) | -0.20 (-0.39, -0.01) | -0.37 (-0.71, -0.04) |
| 5 (most deprived) | 0.002 (-0.043, 0.046) | -0.25 (-0.48, -0.03) | -0.77 (-1.34, -0.21) |

**Table S1(b)** PA facilities accessible by car within 20 minutes

|  | **Urban** | **Small town** | **Rural** |
| --- | --- | --- | --- |
| **Deprivation Quintile** | **Coefficient (99% CI)** | **Coefficient (99% CI)** | **Coefficient (99% CI)** |
| Intercept | -0.06 (-0.37, 0.24) | -0.24 (-0.61, 0.12) | -0.04 (-0.44, 0.35) |
| 2 | -0.02 (-0.06, 0.02) | -0.16 (-0.30, -0.02) | -0.30 (-0.47, -0.12) |
| 3 (middling) | -0.03 (-0.06, 0.01) | -0.18 (-0.30, -0.05) | -0.56 (-0.76, -0.37) |
| 4 | -0.03 (-0.07, -0.001) | -0.11 ((-0.24, 0.02) | -0.59 (-0.86, -0.32) |
| 5 (most deprived) | -0.02 (0.055, 0.065) | -0.16 (-0.32, -0.01) | -0.93 (-1.39, -0.46) |

**Table S1(c)** PA facilities accessible by car within 30 minutes

|  | **Urban** | **Small town** | **Rural** |
| --- | --- | --- | --- |
| **Deprivation Quintile** | **Coefficient (99% CI)** | **Coefficient (99% CI)** | **Coefficient (99% CI)** |
| Intercept | 0.52 (0.12, 0.92) | 0.18 (-0.20, 0.55) | 0.37 (0.04, 0.71) |
| 2 | -0.02 (-0.05, 0.02) | -0.12 (-0.25, 0.001) | -0.26 (-0.42, -0.10) |
| 3 (middling) | -0.02 (-0.05, 0.01) | -0.12 (-0.23, -0.01) | -0.43 (-0.60, -0.25) |
| 4 | -0.03 (-0.06, 0.002) | -0.05 (-0.17, 0.07) | -0.38 (-0.62, -0.13) |
| 5 (most deprived) | -0.02 (-0.05, 0.01) | -0.03 (-0.17, 0.11) | -0.51 (-0.93, -0.09) |
