# Supplementary material for: Access to Recreational Physical Activities by Car and Bus: An Assessment of Socio-Spatial Inequalities in Mainland Scotland
Source: PLoS One. 2013 Feb 7;8(2):e55638. doi: 10.1371/journal.pone.0055638 (PMC3567099; doi:10.1371/journal.pone.0055638)
Supplement: Table S2 — Rate ratio of PA facilities accessible by car within a travel time of 10, 20 and 30 minutes of urban, small town and rural areas by income deprivation. (DOC) [file pone.0055638.s003.doc]

**Table S2 Rate ratio of PA facilities accessible by car within a travel time of 10, 20 and 30 minutes of urban, small town and rural areas by income deprivation**

**Table S2(a)** PA facilities accessible by bus within 10 minutes

|  | **Urban** | **Small town** | **Rural** |
| --- | --- | --- | --- |
| **Deprivation Quintile** | **Coefficient (99% CI)** | **Coefficient (99% CI)** | **Coefficient (99% CI)** |
| Intercept | -5.63 (-5.84, -5.42) | -6.05 (-6.33, -5.77) | -5.70 (-6.14, -5.27) |
| 2 | 0.34 (0.19, 0.48) | 0.37 (0.02, 0.72) | 0.10 (-0.22, 0.43) |
| 3 (middling) | 0.51 (0.37, 0.65) | 0.60 (0.29, 0.92) | 0.43 (0.08, 0.78) |
| 4 | 0.52 (0.39, 0.66) | 0.80 (0.48, 1.13) | 0.63 (0.17, 1.09) |
| 5 (most deprived) | 0.50 (0.37, 0.64) | 0.61 (0.22, 1.00) | 0.36 (-0.43, 1.14) |

**Table S2(b)** PA facilities accessible by bus within 20 minutes

|  | **Urban** | **Small town** | **Rural** |
| --- | --- | --- | --- |
| **Deprivation Quintile** | **Coefficient (99% CI)** | **Coefficient (99% CI)** | **Coefficient (99% CI)** |
| Intercept | -3.38 (-3.68, -3.09) | -3.87 (-4.15, -3.60) | -3.96 (-4.45, -3.47) |
| 2 | 0.28 (0.19, 0.37) | 0.09 (-0.09, 0.26) | -0.07 (-0.34, 0.20_ |
| 3 (middling) | 0.39 (0.30, 0.48) | 0.25 (0.09, 0.41) | 0.27 (-0.03, 0.57) |
| 4 | 0.47 (0.39, 0.55) | 0.39 (0.23, 0.56) | 0.48 (0.06, 0.89) |
| 5 (most deprived) | 0.44 (0.36, 0.52) | 0.36 (0.16, 0.56) | 0.40 (-0.29, 1.10) |

**Table S2(c)** PA facilities accessible by bus within 30 minutes

|  | **Urban** | **Small town** | **Rural** |
| --- | --- | --- | --- |
| **Deprivation Quintile** | **Coefficient (99% CI)** | **Coefficient (99% CI)** | **Coefficient (99% CI)** |
| Intercept | -1.98 (-2.33, -1.64) | -2.27 (-2.64, -1.90) | -2.30 (-2.78, -1.82) |
| 2 | 0.14 (0.07, 0.20) | -0.04 (-0.21, 0.12) | -0.23 (-0.48, 0.03) |
| 3 (middling) | 0.20 (0.13, 0.26) | 0.03 (-0.12, 0.18) | -0.01 (-0.30, 0.27) |
| 4 | 0.25 (0.19, 0.31) | 0.08 (-0.08, 0.24) | 0.13 (-0.27, 0.53) |
| 5 (most deprived) | 0.25 (0.19, 0.31) | -0.01 (-0.20, 0.18) | -0.003 (-0.68, 0.67) |
