# Supplementary material for: Access to Recreational Physical Activities by Car and Bus: An Assessment of Socio-Spatial Inequalities in Mainland Scotland
Source: PLoS One. 2013 Feb 7;8(2):e55638. doi: 10.1371/journal.pone.0055638 (PMC3567099; doi:10.1371/journal.pone.0055638)
Supplement: Table S3 — Median compound accessibility by income deprivation and urban/rural classification. (DOC) [file pone.0055638.s004.doc]

**Table S3 Median compound accessibility by income deprivation and urban/rural classification**

| **URC** | **Deprivation Quintile** | **Median Compound Accessibility** | | |
| --- | --- | --- | --- | --- |
|  |  | 10 minutes | 20 minutes | 30 minutes |
| **Urban** | 1 (most affluent) | 471.13 | 1609.89 | 2656.86 |
|  | 2 | 382.62 | 1377.29 | 2366.52 |
|  | 3 (middling) | 299.42 | 1189.79 | 2149.91 |
|  | 4 | 258.38 | 1012.47 | 1896.35 |
|  | 5 (most deprived) | 240.32 | 784.42 | 1417.18 |
| **Small town** | 1 (most affluent) | 118.67 | 605.80 | 1222.06 |
|  | 2 | 75.39 | 409.49 | 956.83 |
|  | 3 (middling) | 51.92 | 202.95 | 662.10 |
|  | 4 | 46.45 | 298.51 | 992.70 |
|  | 5 (most deprived) | 42.90 | 325.92 | 1128.47 |
| **Rural** | 1 (most affluent) | 62.89 | 408.97 | 903.43 |
|  | 2 | 39.74 | 194.76 | 427.59 |
|  | 3 (middling) | 28.52 | 119.42 | 254.72 |
|  | 4 | 61.27 | 340.53 | 783.26 |
|  | 5 (most deprived) | 21.06 | 130.18 | 408.48 |
